# Short-term calcium peroxide application promotes soil microbial interactions to improve peanut yield in acidic soils

**DOI:** 10.3389/fmicb.2026.1780622

**Published:** 2026-03-30

**Authors:** Yunfei Yu, Xiaoxue Li, Azhar Sohail Shahzad, Shunyao Zhuang

**Affiliations:** 1 State Key Laboratory of Soil and Sustainable Agriculture, Institute of Soil Science, Chinese Academy of Sciences, Nanjing, China; 2 University of the Chinese Academy of Sciences, Beijing, China

**Keywords:** calcium peroxide, hydrogen peroxide, network interactions, pathogen, peanut

## Abstract

The deterioration of soil microorganisms stands as a key barrier to the sustainable cultivation of peanut. Peroxides such as hydrogen peroxide (H_2_O_2_) and calcium peroxide (CaO_2_) with antimicrobial efficacy may inhibit soil pathogens and improve microbial community, but their effects on the soil properties, microbiome and peanut yield are still unclear. In this study, a field experiment was conducted to investigate the effects of CaO_2_ (CP, with application rate of 4.5 t ha^−1^) and H_2_O_2_ (HP, with application rate of 22.5 t ha^−1^) and no peroxide application (CK) on physicochemical properties, microbial communities and network structures of acidic soils in which peanut had been cultivated for 13 years. Compared with CK, calcium peroxide application significantly increased soil pH. Hydrogen peroxide showed a similar trend, but not significant. Principal co-ordinates analysis (PCoA) indicated that peroxides application significantly affected the fungal community structure, while it had a weaker impact on the bacterial community. With peroxides application, soil harmful fungus *Fusarium* and the fungal functional group associated with plant pathogen declined. Network co-occurrence analysis showed that peroxides enhanced bacterial network interactions, with bacterial stability increasing markedly by 2.19- and 1.40-fold in calcium peroxide and hydrogen peroxide treatments, respectively. Peanut yield was correlated with the microbial network properties, and significantly increased by 31.7% in calcium peroxide. Random forest model analysis further revealed that bacterial stability, soil pH, bacterial negative cohesion, and complexity were key factors for peanut yield. In conclusion, short-term calcium peroxide application increased soil pH and bacterial stability, thereby promoting peanut yield in acidic soils.

## Introduction

Peanut (*Arachis hypogaea* L.) is an essential economic and oil crop cultivated globally, for its substantially growing demand over the past decades (Bertioli et al., 2016; Sobolev et al., 2013). However, long-term peanut monocropping can cause an imbalance in soil microbial communities, resulting in severe continuous cropping obstacles and ultimately a reduction in peanut yield (Li et al., 2014a). Although crop rotation can alleviate continuous cropping obstacles by improving the soil environment and reshaping microbial communities (Wu et al., 2025; Zou et al., 2023), long-term crop rotation may also lead to homogenization of soil bacterial community, potentially diminishing its initial beneficial effects (Maier et al., 2018; Navarro-Noya et al., 2013). Microorganisms play a crucial role in regulating soil ecological functions and supporting crop growth and health (Maier et al., 2018); thus, healthy soil typically exhibits a more stable microbial community structure to resist pathogens (Liu et al., 2015). However, the diversity and function of soil microorganisms tend to decline in long-term continuous cropping or crop rotation (Navarro-Noya et al., 2013; Yu et al., 2024). Therefore, understanding the changes of soil microbiome and their interactions is essential for revealing the potential mechanisms of long-term cultivation obstacles in peanut production.

Microbial deterioration is a profound consequence of long-term monocropping, posing severe threats to soil functionality and crop health (Feng et al., 2022; Liu et al., 2019). Long-term peanut cultivation significantly reduced both microbial richness and diversity (Zou et al., 2023). With prolonged peanut cultivation, the relative abundance of beneficial bacteria such as *Candidatus_Entotheonella, Bacillus* and *Bryobacter* decreased, while the detrimental fungi like *Fusarium, Colletotrichum*, and *Metarhizium* enriched in soils (Chen et al., 2020; Yu et al., 2024). The accumulation of pathogenic microorganisms elevates the risk of soil-borne pathogens outbreaks (Wang et al., 2025). Moreover, long-term continuous cropping can alter soil physicochemical properties, further affecting microbial community structure (Li et al., 2022; Zhu et al., 2022). Recent studies have indicated that continuous cropping led to a decrease in soil pH (Shen et al., 2019; Xu et al., 2024), which in turn simplifies the interaction network of bacteria and fungi (Yu et al., 2024). Consequently, the reduced stability and complexity of microbial communities under continuous cropping tend to weaken the resistance of microorganisms to pathogens, thereby intensifying plant disease incidence and reducing crop yields (Arunrat et al., 2023; Gu et al., 2022). Thus, it is crucial to regulate the soil microbial community through specific intervention measures to alleviate soil cultivation obstacles and increase peanut yields.

Calcium peroxide and hydrogen peroxide have been widely recognized as environmentally friendly and safe oxidizing and sterilizing agents commonly applied in soil improvement, crop cultivation, and environmental disinfection (Apul et al., 2022; Lu et al., 2017; Mei et al., 2017; Szpunar-Krok et al., 2020). Their primary mechanism is to introduce reactive oxygen species to disinfect and sterilize environmental microorganisms, while simultaneously decomposing and releasing oxygen to improve the environmental conditions (McDonnell and Russell, 1999). Recent studies have indicated that hydrogen peroxide can be used to relieve continuous cropping obstacles. For instance, Xu et al. (2023) demonstrated that hydrogen peroxide application inactivated part of the *Fusarium* population and simultaneously increased the relative abundance of beneficial microbes like *Bacillus, Mortierella*, and *Guehomyces* in apple replanted soil, thereby relieving apple replant disease. Meanwhile, calcium peroxide can also increase soil pH. Previous studies have indicated that lime application can improve soil acidification in peanut monocropping soils, which in turn reshapes microbial community composition, thereby improving soil health and peanut growth (Lu et al., 2023; Yang et al., 2024). Therefore, it is hypothesized that calcium peroxide may have similar or better effects in acidic soils. However, the potential associations among hydrogen peroxide and calcium peroxide application, soil physicochemical properties, microbial communities, and peanut growth require further investigation.

Therefore, in this study, we conducted a field experiment to explore the effects of hydrogen peroxide and calcium peroxide application on soil properties, microbial communities, and peanut yield under long-term cropping, as well as the potential relationship among their changes. In a field with 13-year continuous peanut cultivation, we performed a short-term peroxide addition experiment and investigated the dynamics of bacterial and fungal communities by amplicon sequencing. Besides, soil physicochemical properties and peanut yields were measured. The objectives of this study were to (1) evaluate the impacts of calcium peroxide and hydrogen peroxide on soil properties, microbes, and peanut yield, (2) clarify the potential associations among soil properties, bacterial and fungal interaction networks, and peanut yield.

## Materials and methods

### Study site

The field experiment was performed at the Yingtan National Agricultural Ecosystem Observation and Research Station in Jiangxi Province, China (28°12′N, 116°55′E). The site has a typical subtropical climate with an average annual precipitation of 1,881.8 mm and an average annual temperature of 18.4 °C. The experimental field has been cultivating peanuts (*Arachis hypogaea*, a legume) since 2010. The field soil used in the experiment is classified as loamy clay derived from Quaternary red clay (Ferric Acrisols in the FAO classification system). In the upper 20 cm of the soil profile, the selected properties of soil include pH of 5.96, organic carbon (SOC) content of 10.11 g kg^−1^, total nitrogen (TN) of 0.68 g kg^−1^, total phosphorus (TP) of 0.85 g kg^−1^, available phosphorus (AP) of 41.36 mg kg^−1^, and available potassium (AK) of 385.0 mg kg^−1^.

### Field experimental design

The experiment was conducted from March to August 2023 during the peanut-growth season and included the following three treatments: (i) CK, control without peroxide addition; (ii) CP, 0.2% calcium peroxide application (4.5 t ha^−1^), and (iii) HP, 1% hydrogen peroxide application (22.5 t ha^−1^). The calcium peroxide (calcium carbonate and calcium peroxide content ≥60%) and hydrogen peroxide (with a concentration of 30%) were purchased from Jiangmen Nongxin Chemical Co., Ltd. (Jiangmen City, Guangdong Province, P.R. China) and Zhenjiang Jiuyi Chemical Co., Ltd. (Zhenjiang City, Jiangsu Province, P.R. China), respectively. Fifteen days prior to peanut sowing, the calcium peroxide powder was incorporated into the topsoil, hydrogen peroxide was sprayed onto the topsoil, and then sterile water was applied to adjust soil moisture consistently. In addition, all treatments were treated with urea at 130 kg N ha^−1^, superphosphate at 75 kg P_2_O_5_ ha^−1^, and potassium chloride at 180 kg K_2_O ha^−1^ for each peanut season. The experiment was conducted using a fully randomized layout with three repetitions and each plot was designed with an area of 3 × 5 m^2^ separated by an earth ridge. A locally cultivated peanut variety “Ganhua No.1” was used as the test crop, sown on 10 April 2023 and harvested on 8 August, with field management conducted according to local planting practices. The peanut plant height was measured on June 13, 2023, and the yield was determined at harvest on August 12, 2023.

### Soil sampling and analysis

Soil samples near the root were collected on the 15th (April 10), 80th (June 13) and 140th (August 12) days after peroxides treatment, corresponding to the germination, flowering and harvest stages of peanut, respectively. Specifically, three peanut plants were randomly selected from each experimental area, and sterile soil shovels were used to carefully excavate the topsoil layer (0–20 cm) around the root systems as root-zone soil samples. The root-zone soil collected from the three plants was thoroughly mixed to form a composite sample, which was then placed in a sterile polyethylene bag and transported to the laboratory. A total of 27 samples (3 treatments × 3 replicates × 3 sampling times) were processed by removing impurities, sieving through a 2-mm mesh, and prepared for physicochemical and biological analyses. Each mixed soil sample was divided into three parts: one was immediately stored at −80 °C for DNA extraction; another was kept at 4 °C for measurements of nitrate (${\text{NO}}_{3}^{-}$-N) and ammonium nitrogen (${\text{NH}}_{4}^{+}$-N) concentrations; the remaining portion was air-dried for the evaluation of pH (1:2.5 H_2_O), SOC, TN, TP, AP, and AK following the methods described.

### Microbial community composition analysis

All soil samples were detected for the analysis of bacterial and fungal community composition by the Illumina MiSeq high-throughput sequencing method. Total DNA was extracted from 0.5 g of soil using the FastDNA SPIN Kit (MP Biomedicals, USA). The bacterial 16S rRNA gene (338F-806R) and fungal ITS region (ITS1-1737F, ITS2-2043R) were amplified and sequenced using the Illumina MiSeq paired-end 300 bp platform (Illumina Corporation, USA). Only sequences with lengths of 200–579 bp for bacteria and 200–535 bp for fungi were retained to calculate Operational Taxonomic Units (OTUs) at 97% similarity using UPARSE. OTUs were taxonomically classified using the classify-sklearn naive Bayes classifier, referencing the Silva database (version 13.8) for bacteria and the UNITE database (version 8.3) for fungi. A total of 2,030,825 and 2,070,084 high-quality bacteria and fungi sequences, respectively, were obtained from the 27 soil samples and were clustered into 11,934 bacteria and 2,353 fungi OTUs. In addition, fungal OTUs were categorized into trophic groups (saprotrophs, pathogens, and mycorrhiza) by the FUNGuild tool (Nguyen et al., 2016), with only “highly probable” and “probable” classifications considered. The raw sequence data have been submitted to the National Center for Biotechnology Information (NCBI) Sequence Read Archive under the Bioproject PRJNA1311041 and Bioproject PRJNA1311036.

### Microbial network construction

Microbial network analysis was conducted to explore the occurrence patterns of bacterial communities, fungal communities, and interkingdom interactions based on correlations. The analysis was limited to OTUs with relative abundances of >0.01%, and then all pairwise Spearman correlations between OTUs were computed, and only correlations with a Spearman's coefficient >0.7 and a *p* < 0.05 were retained for bacterial and fungal networks construction (Fan et al., 2019; Han et al., 2022). Moreover, the OTU tables were rarefied for interkingdom networks between bacteria and fungi. Specifically, the OTUs with relative abundances of >0.1% were selected for analysis, and the correlations with a Spearman's coefficient >0.65 and a *p* < 0.05 were retained for interkingdom networks construction. A set of metrics including average degree, modules, modularity, average path length, and cohesion was calculated using the “microeco” R package (Liu et al., 2021) to describe the network. In the co-occurrence networks of bacteria and fungi, nodes represent OTUs, and edges represent Spearman associations between nodes. Moreover, the networks were visually represented using Gephi version 0.9.2 (http://gephi.github.io/).

### Network complexity and stability based on cohesion index

Cohesion refers to the degree of association among taxa, driven by both positive and negative species interactions, as well as similarities or differences in their ecological niches. By separately assessing positive and negative co-occurrences, cohesion provides insights into the structure and dynamics of microbial communities. For each sample (*j*), positive and negative cohesion values are calculated as the sum of significant positive or negative correlations between taxa, weighted by their abundances (Herren and McMahon, 2017; Yuan et al., 2021):


$$\begin{array}{lll} C_{j}^{pos} & = & \overset{n}{\underset{i=1}{\sum }}a_{i}\cdot {\bar{u}}_{i,u>0}\left(Positive\;Cohesion\right)\\ C_{j}^{neg} & = & \overset{n}{\underset{i=1}{\sum }}a_{i}\cdot {\bar{u}}_{i,u<0}\left(Negative\;Cohesion\right) \end{array}$$

where *a*_*i*_ represents the abundance of taxon *i* in sample *j*, and ${\bar{u}}_{\text{i},\text{r}>0}$ and ${\bar{u}}_{\text{i},\text{r}<0}$ are positive and negative connectedness, respectively. For a given taxon *i* within a network, the positive (${\bar{u}}_{\text{i},\text{r}>0}$) and negative connectedness (${\bar{u}}_{\text{i},\text{r}<0}$) were calculated as the mean values of all significant positive and negative correlations, respectively, between taxon *i* and other microbial taxa in the network.

The complexity of microbial network was calculated by the sum of positive cohesion and the absolute value of negative cohesion as follows (Hernandez et al., 2021):


$$\begin{array}{l} Complexity=positive\;cohesion+|negative\;cohesion| \end{array}$$

The stability of the microbial network was determined according to the ratio of negative to positive associations between taxa:


$$\begin{array}{l} Stability=\frac{\left|negative\;cohesion\right|}{positive\;cohesion} \end{array}$$

## Statistical analyses

Statistical comparisons among different soil treatments were conducted via one-way analysis of variance (ANOVA), followed by Duncan's multiple-range test at the 95 % confidence level. The threshold for all statistical significance was set at *p* < 0.05. Chao1 index calculated from the rarefied feature table was used to evaluate the α-diversities of bacteria and fungi. Principal coordinates analysis (PCoA) based on Bray-Curtis distance was performed to examine the β-diversities of bacteria and fungi, while permutational multivariate analysis of variance (PERMANOVA) was employed to assess the significance of microbial community differences among various treatments and sampling times. Redundancy analysis (RDA) coupled with hierarchical partitioning was conducted to explore the associations between microbial communities and soil properties using the R “rdacca.hp” package (Lai et al., 2022). Spearman's correlation coefficient was used to evaluate the relationships between microbial community composition and soil properties, as well as the associations among microbial network properties, peanut yield, and soil properties. The random forest model was constructed with the following parameters: importance = TRUE, ntree = 500, and nrep = 1,000 to evaluate the relative importance of predictor variables for peanut yield, using the R “rfPermute” package (Liao et al., 2024).

## Results

### Soil physicochemical properties, microbial composition and diversity

**Figure 1:**
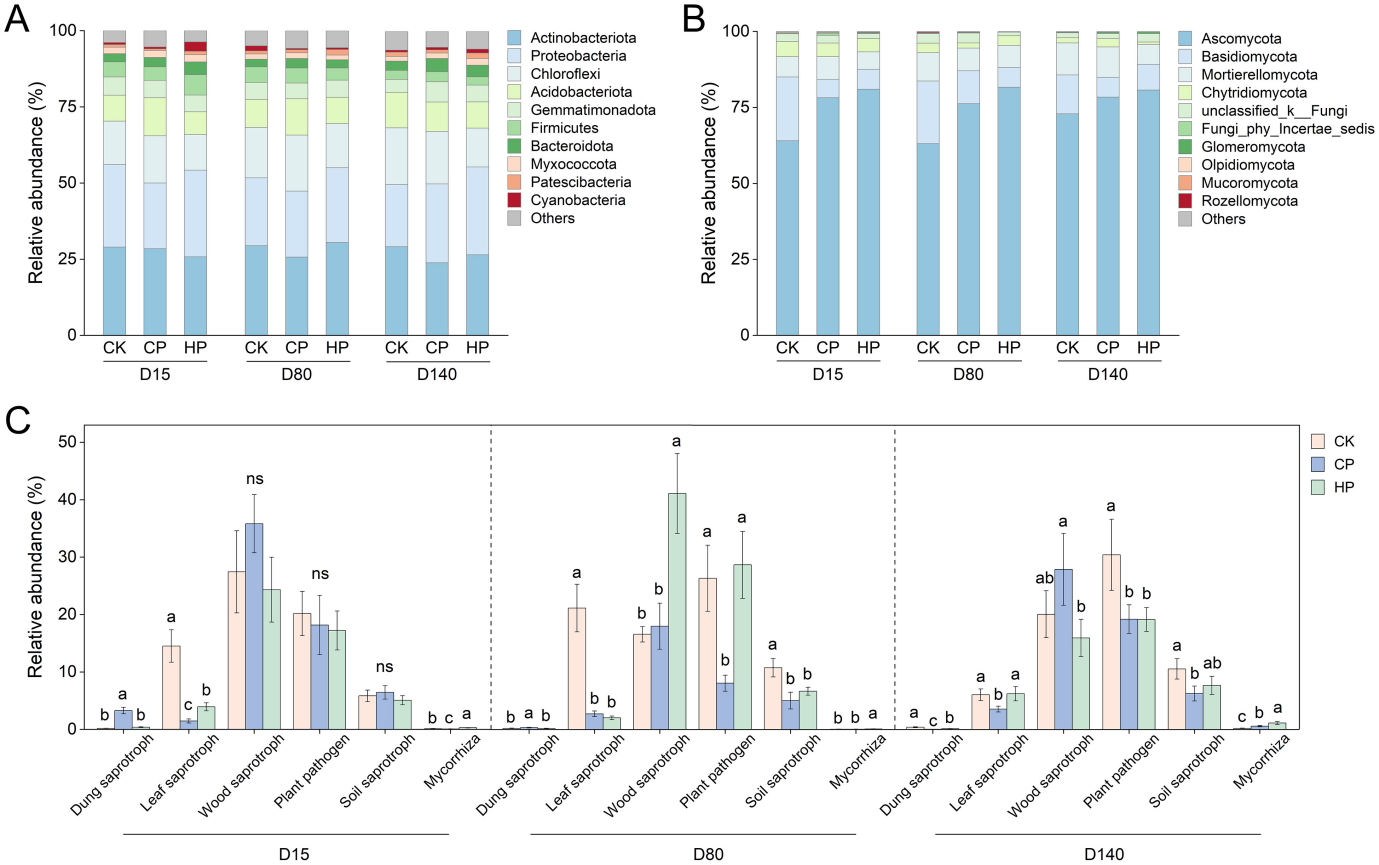
Relative abundance of bacterial **(A)**, fungal **(B)** phyla and fungal functional guilds **(C)** in different treatments (*n* = 3). Different letters represent the significant difference at *p* < 0.05 by Duncan's test. Treatment: CK, control without peroxide application; CP, 0.2% calcium peroxide application; HP, 1% hydrogen peroxide application.

The temporal dynamics of soil physicochemical properties were presented in Supplementary Table S1 across plant development under different treatments. In brief, the soil treated with peroxides application exhibited significantly higher pH and lower available K than the CK treatment, especially CP treatment. Compared with the CK treatment, ${\text{NH}}_{4}^{+}$ in CP treatment significantly decreased at the early plant developmental stage (days 15) and ${\text{NO}}_{3}^{-}$ reduced at the later stage (days 140). The HP treatment displayed similar trends. Compared with CK, there was no significant difference in bacterial α-diversity between the CP and HP treatments (Supplementary Figure S1A), while the fungal α-diversity of CP treatment showed a significant increase at the early stage (Supplementary Figure S1B). There were no significant differences in bacterial community composition (Supplementary Figure S2A, PERMANOVA test: *R*^2^ = 0.331, *p* = 0.339 for treatment; *R*^2^ = 0.142, *p* = 0.067 for sampling stage); however, soil fungal community compositions showed significant differences among the three treatments (Supplementary Figure S2B, PERMANOVA test: *R*^2^ = 0.212, *p* = 0.028). Additionally, the relative abundance of *Proteobacteria* decreased under CP treatment compared to CK at early stage (Figure 1A). There was an enrichment of the fungal phylum *Ascomycota* in peroxide treatments (Figure 1B), while the relative abundance of fungus *Fusarium* reduced at later stage (Supplementary Figure S3B). Moreover, fungal functional groups associated with plant pathogen decreased in CP and HP treatments at later stage (Figure 1C). In addition, leaf saprotroph decreased in CP across stages, whereas mycorrhiza increased in HP relative to CK.

### Relationships between soil physicochemical properties and microbial community structure

**Figure 2:**
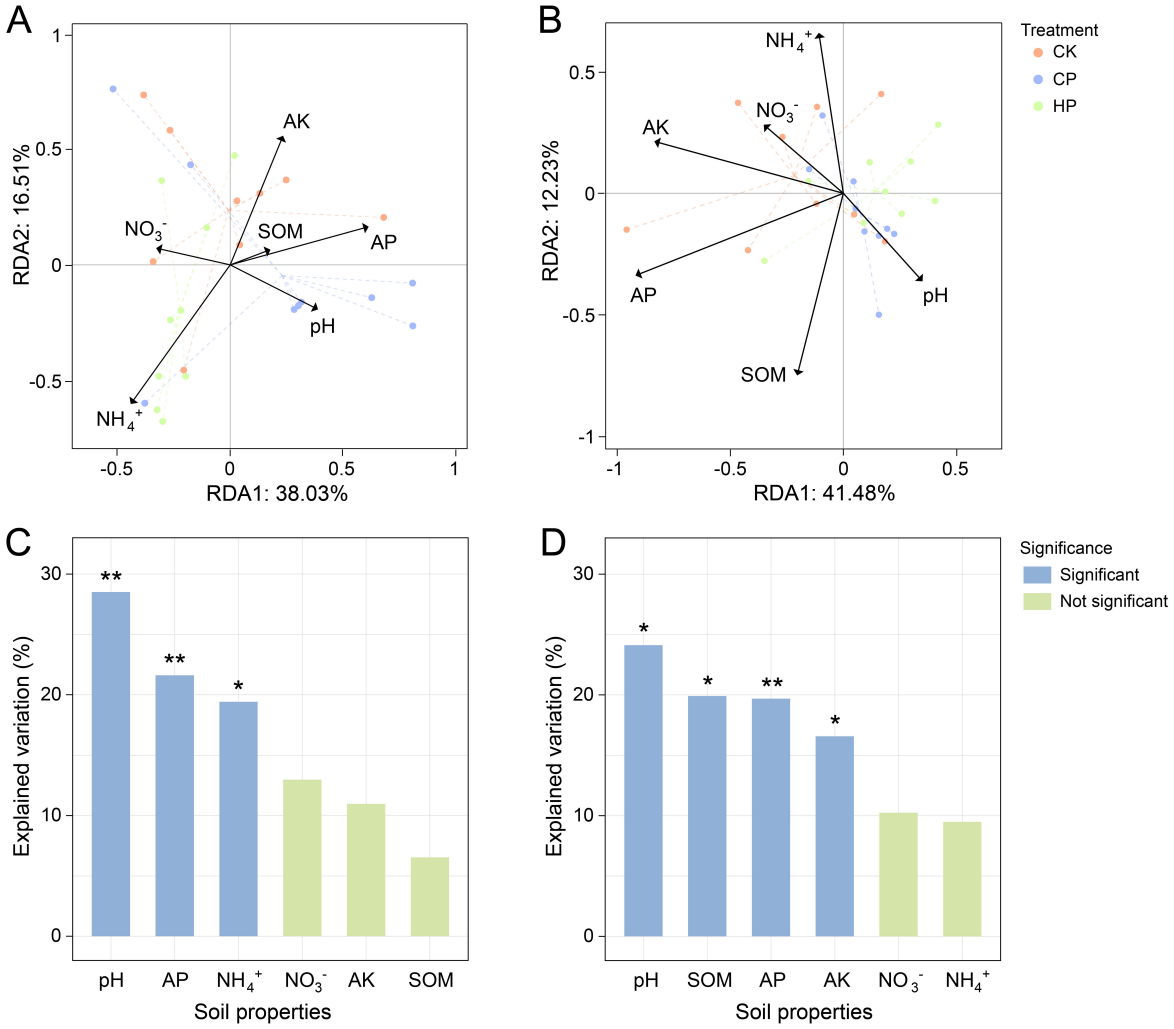
Redundancy analysis (RDA) presenting the relationships between soil physicochemical properties and the microbial community structure of bacteria **(A)**, fungi **(B)**. Hierarchical partitioning indicating the proportion of environmental factors explaining the microbial community of bacteria **(C)** and fungi **(D)**. *p < 0.05; **p < 0.01.

RDA diagrams revealed the effects of soil physicochemical properties on the microbial community. RDA1 and RDA2 accounted for 38.03 and 16.51% of the total variance in the bacterial community, and 41.48 and 12.23% of the variance in the fungal community based on relative abundance of phyla, respectively (Figures 2A, B). Hierarchical partitioning indicated that soil pH, AP and ${\text{NH}}_{4}^{+}$ were the primary factors significantly influencing the bacterial community structure, accounting for 28.51%, 21.61%, and 19.41% of the explained variation, respectively (Figure 2C). Soil pH, SOM, AP and AK were the dominant factors affecting the fungal community, and corresponded to 24.12%, 19.90%, 19.68%, and 16.57% of the explained variation (Figure 2D). The correlation heatmap further established the relationships between soil physicochemical properties and microbial community composition. Soil pH was positively correlated with the relative abundance of *Bacteroidota* but positively correlated with *Patescibacteria* at the bacterial phylum (Supplementary Figure S4A). The ${\text{NH}}_{4}^{+}$ exhibited a positive correlation with *Proteobacteria* but a negative correlation with *Acidobacteriota* and *Chloroflexi*. Additionally, soil AP and AK showed a positive correlation with the fungal phylum *Ascomycota* but a negative correlation with *Basidiomycota* (Supplementary Figure S4B).

### Microbial network associations in soils with different treatments

**Figure 3:**
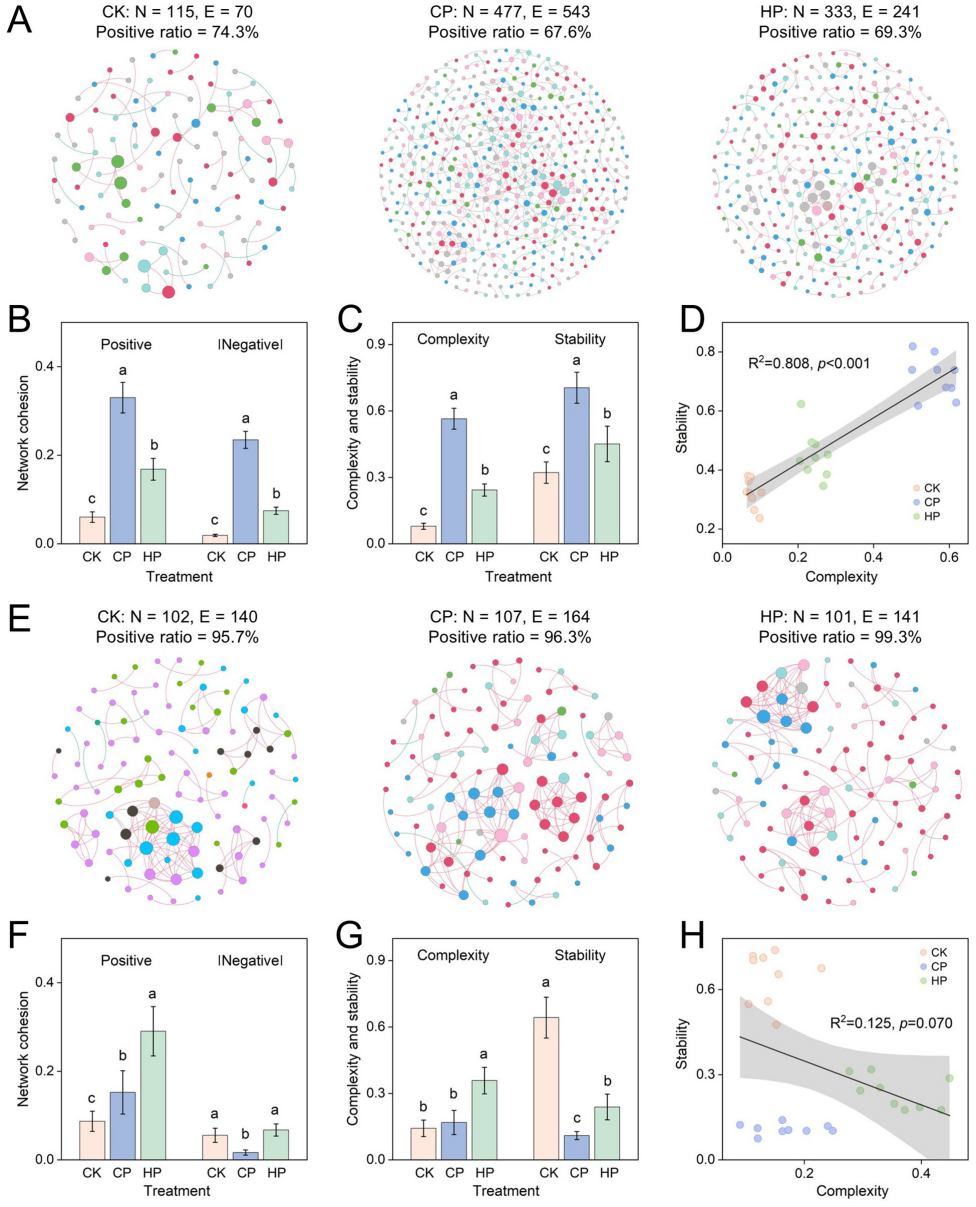
The co-occurrence network of bacteria **(A)** and fungi **(E)** in different treatments (calculated on the three sampling stages, *n* = 9). Network cohesion **(B, F)**, complexity and stability **(C, G)** and regression relationships between the network complexity and stability **(D, H)** of bacteria and fungi. Different colors in microbial networks indicate different phyla. Different letters represent the significant difference at *p* < 0.05 by Duncan's test. Treatment: CK, control without peroxide application; CP, 0.2% calcium peroxide application; HP, 1% hydrogen peroxide application.

Co-occurrence networks were constructed to depict the complex interactions within microbial community. In general, the bacterial networks under peroxide treatments both displayed a marked increase in the number of nodes and edges compared to CK, among which CP treatment was the highest with 477 nodes and 543 edges (Figure 3A). Additionally, various topological features, including negative associations, average degree, network diameter, average path length, and heterogeneity consistently exhibited increases under peroxide treatments (Supplementary Table S2). However, similar results were not observed in fungal networks (Figure 3E), suggesting that peroxides have a significantly weaker effect on fungal network associations compared to bacterial ones (Supplementary Table S3).

Bacterial and fungal networks exhibited divergent patterns of complexity and stability across treatments. The positive cohesion of bacterial network increased from 0.06 of CK treatment to 0.33 of CP, and 0.17 of HP, while the |negative cohesion| similarly rose from 0.02 of CK treatment to 0.24 of CP, and 0.08 of HP (Figure 3B). The complexity of bacterial network under CP and HP treatments was 7.12- and 3.06-fold higher than that under CK, respectively (Figure 3C). The stability showed the same trend of change, with the highest stability being CP treatment. Additionally, linear regression further established that the complexity was significantly positively correlated with the stability of bacterial network (Figure 3D, *R*^2^ = 0.808, *p* < 0.001). However, fungal network cohesion exhibited different results. We found the positive cohesion of fungal network in CP and HP soils was 1.75- and 3.36-fold higher than that in CK, respectively, while the |negative cohesion| in CP was 3.35-fold lower than that in CK (Figure 3F). Correspondingly, compared to CK, although HP treatment increased the complexity of fungal network, its stability was significantly reduced, and CP showed the same trend (Figure 3G). Furthermore, no linear correlation was observed between the complexity and stability of the fungal network (Figure 3H, *R*^2^ = 0.125, *p* = 0.070).

### Interkingdom interaction between bacteria and fungi

**Figure 4:**
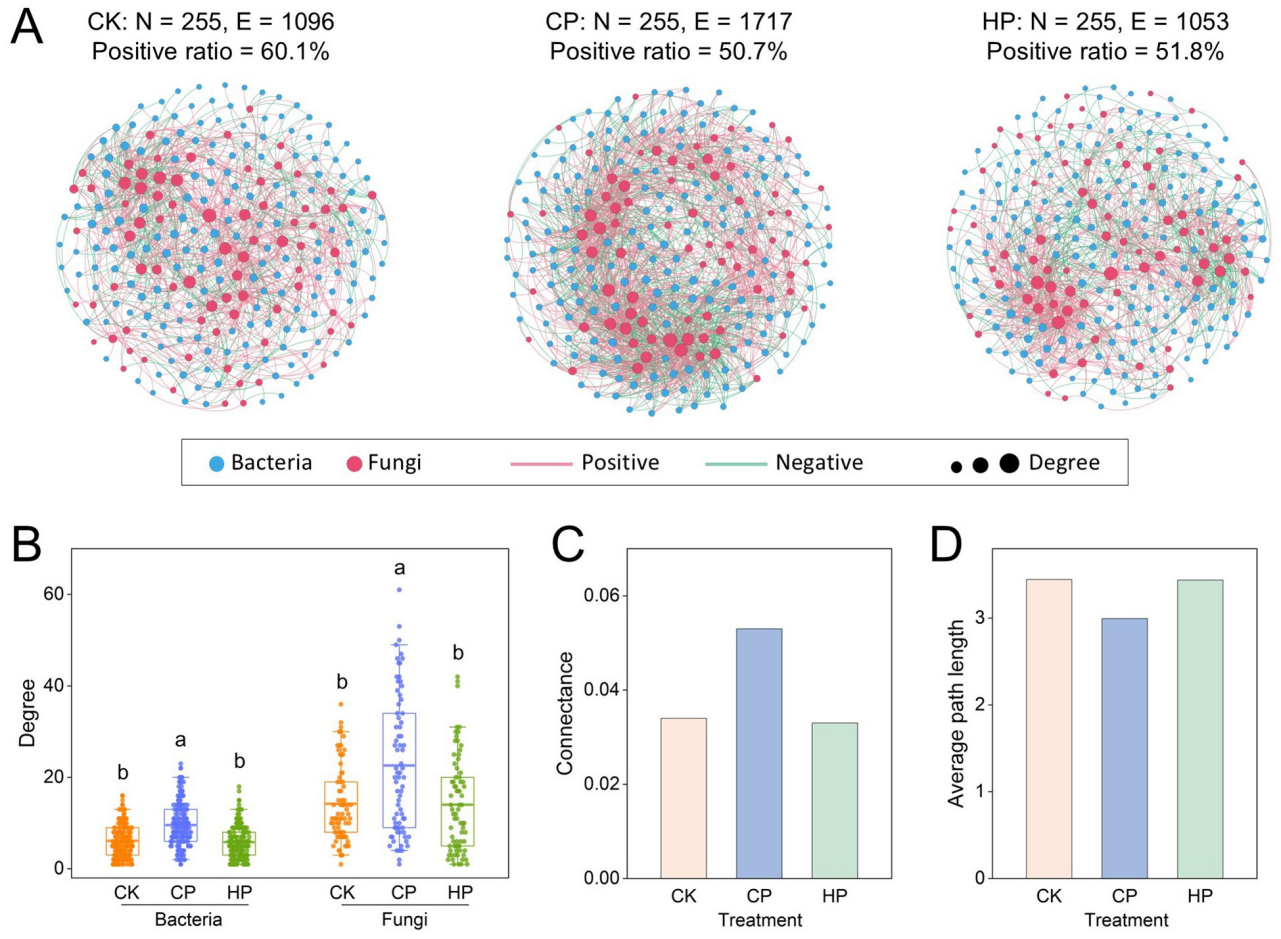
Interkingdom co-occurrence networks of bacteria and fungi constructed for different treatments **(A)**. Degree values for bacteria and fungi in the interkingdom networks **(B)**. Connectance **(C)** and average path length **(D)** of the networks. Different letters represent the significant difference at *p* < 0.05 by Duncan's test. Treatment: CK, control without peroxide application; CP, 0.2% calcium peroxide application; HP, 1% hydrogen peroxide application.

Elucidating bacterial-fungal interactions is critical for understanding microbial function in soil ecosystem. It was observed that 180 bacterial nodes and 75 fungal nodes were engaged in interactions across the three treatments (Figure 4A). Notably, CP treatment markedly increased the edges in the interdomain network, reaching 1,717, compared to 1,096 in the CK. The predominant interaction under CK was cooperative, with a positive correlation ratio of 60.1%. However, the positive correlation ratio of interdomain interactions declined to 50.7% under CP and 51.8% under HP. In CP treatment, the average degree of connectivity for bacteria and fungi were respectively 1.57- and 1.59-fold higher than those in CK (Figure 4B). Additionally, we observed the interdomain connectance in CP improved by 55.9%, while the average path length decreased 13.1%, compared to CK (Figures 4C, D). Furthermore, although we detected that the modularity and heterogeneity of the interdomain network in CP declined by 17.4 and 18.1% compared with CK, respectively, similar trend was not observed in HP (Supplementary Table S4).

### Importance of microbial networks in regulating soil properties and crop yield and the key environmental drivers

**Figure 5:**
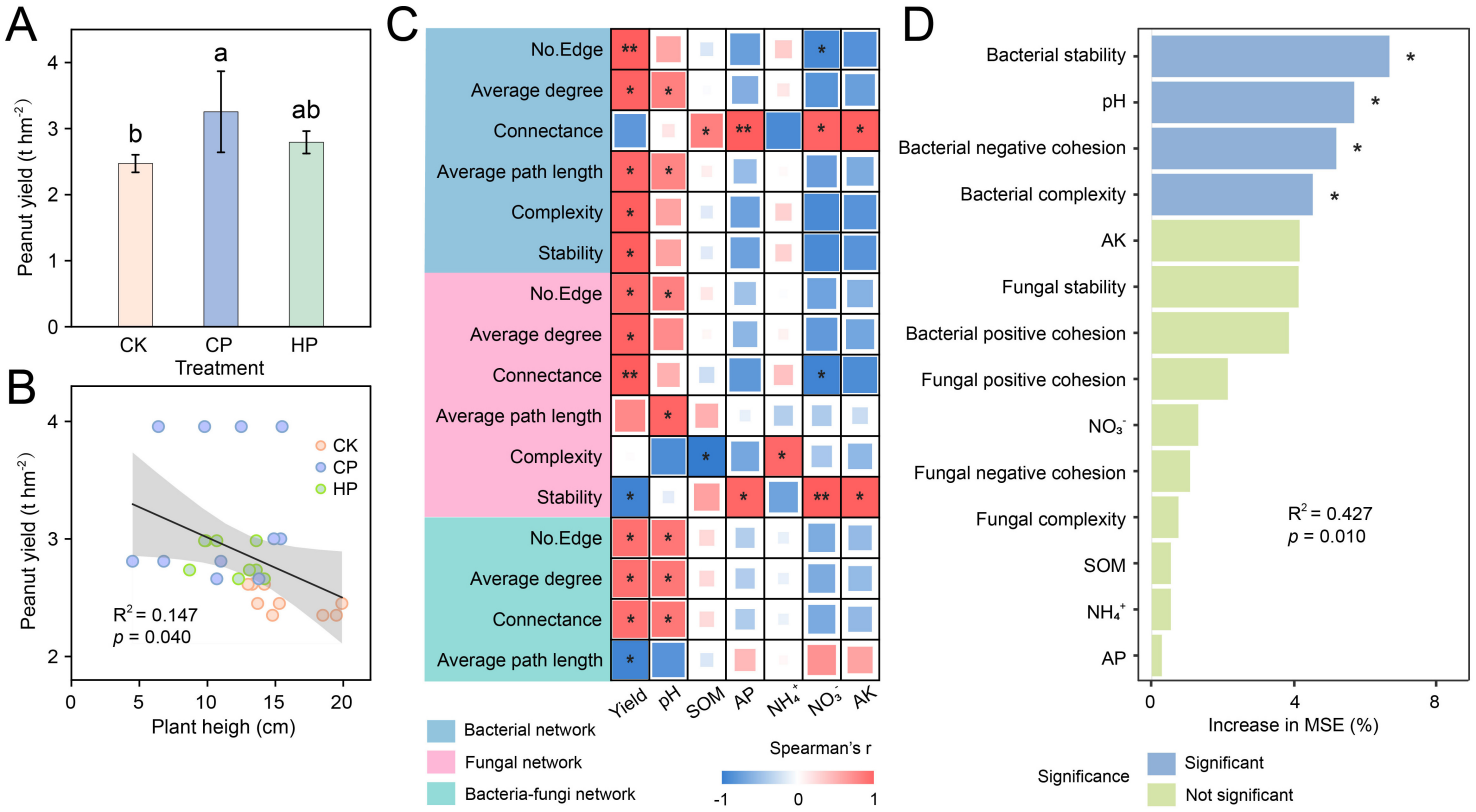
Peanut yield **(A)**, regression relationships between the yield and plant heigh of peanut **(B)**. Spearman correlation between microbial networks and crop yield and soil properties **(C)**. Relative importance of the principal components revealing the dominance of microbial network and soil physicochemical properties on the peanut yield based on random forest model analysis **(D)**. *p < 0.05; **p < 0.01.

Compared with CK, peanut yield was significantly increased by 31.7% under CP treatment, whereas it increased by 13.1% under HP treatment without reaching statistical significance (Figure 5A). Interestingly, linear regression analysis indicated that the peanut yield was significantly and negatively correlated with the plant height (Figure 5B, *R*^2^ = 0.147, *p* = 0.040). In general, compared to soil physicochemical properties which showed relatively weak correlations with the network indices of bacteria and fungi, the peanut yield displayed a close relationship with bacterial, fungal, and bacteria-fungi network indices (Figure 5C). Specifically, the peanut yield was positively correlated to the edges and average degree of all networks, as well as the average path length, complexity, and stability of bacterial network, and connectance of fungal and bacteria-fungi networks. Meanwhile, the stability of fungal network and average path length of bacteria-fungi network were negatively correlated with the peanut yield. The established random forest model revealed the relative importance of the principal components of microbial networks and soil physicochemical properties with respect to peanut yield (Figure 5D, *R*^2^ = 0.427, *p* = 0.010). The stability, negative cohesion, and complexity of bacterial community were significant and influential factors affecting the peanut yield. Among the soil physicochemical properties, pH emerged as the most significant and influential predictor for peanut yield.

## Discussion

### Effects of peroxides on soil physicochemical properties and microbial community structure

The soil obstacles in long-term peanut cultivation are primarily related to the cumulative deterioration of soil physicochemical properties and the consequent disorganization of microbial community structure (Li et al., 2014b, 2019; Yu et al., 2024). The application of calcium peroxide elevated soil pH (Supplementary Table S1), suggesting that it may ameliorate soil properties to an extent comparable with lime (Lu et al., 2023; Zhang et al., 2022). However, the increase in acidic soil pH can reduce the solubility of potassium, and the abundant Ca^2+^ introduced by calcium peroxide inputs could trigger cation competition: Ca^2+^ may displace K^+^ from soil colloid exchange sites, promoting K^+^ entrapment within mineral lattices and consequently diminishing potassium availability (Han et al., 2023). Additionally, calcium peroxide could enhance the fixation and volatilization of ammonium, causing an initial decline in NH4^+^ and, consequently, a later reduction in ${\text{NO}}_{3}^{+}$ (Tadesse et al., 2025). Peroxides can alter soil microbial communities through both direct disinfection and indirect effects mediated by shifts in soil physicochemical properties (Chen et al., 2023; Kakosová et al., 2017).

Soil fungal community was more sensitive to peroxide application than bacterial community (Supplementary Figure S2). Peroxide treatments consistently enriched *Ascomycota* and the fungal functional groups associated with leaf saprotroph and mycorrhiza, suggesting that peroxides may stimulate these microorganisms to promote nutrient cycling (Li et al., 2024). Moreover, peroxides suppressed the pathogenic genus *Fusarium* and reduced the proportion of plant pathogen-associated fungal functional groups at the late growth stage. This pathogen inhibition is likely mediated by peroxide-induced improvements in soil microbial community structure, thereby enhancing plant defense responses to soil pathogenic fungi (Xu et al., 2023).

Although peroxide treatments did not significantly alter all soil physicochemical properties, they may still exert a marked impact on microbial community structure (Philippot et al., 2024). For example, even mild changes in SOM could significantly influence fungal community (Figure 2D), likely due to the physical protection of SOM by Ca^2+^, which further affected the fungal decomposition process (Shabtai et al., 2023; Underwood et al., 2024). Specifically, owing to different ecological strategies (Wang and Kuzyakov, 2024), bacteria are predominantly copiotrophic and sensitive to soluble nutrients (e.g., ${\text{NH}}_{4}^{+}$) (Li R. C. et al., 2023; Wang et al., 2024), while fungi are primarily oligotrophic, displaying greater responsiveness to recalcitrant or adsorbed nutrients (e.g., K) (Fang et al., 2025). Moreover, consistent with previous studies, soil pH emerged as the primary environmental factor influencing microbial community structure (Duan et al., 2025). Additionally, AP, significantly impacted by soil pH, may also exert substantial effect on microbial communities (Cao et al., 2024; Wu et al., 2022). Notably, despite soil nutrients were generally abundant in our study, they should be promptly supplemented by fertilization when applying peroxides in practice to prevent potential nutrient depletion and support crop growth.

### Effects of peroxides on microbial network associations

Cohesion parameters were used to assess the strength of the positive and negative interactions within bacterial and fungal community, along with the overall complexity and stability of the communities (Hernandez et al., 2021; Herren and McMahon, 2017). The co-occurrence networks of bacteria under peroxides suggested that the strength of interactions enhanced and the tightness of the interconnection between colonies in bacterial community increased. Under peroxide treatments, increased positive cohesion indicated more cooperative relationships such as symbiosis, cross-feeding, and commensalism among co-existing taxa with similar niches (Shi et al., 2016), enabling microbiota to survive cooperatively under changing environmental conditions (Ma et al., 2020; Zhang et al., 2024). Additionally, the prevalence of negative cohesion reflected the potential for competitive behaviors that promoted microbial niche partitioning and enhanced environmental adaptability (Wu et al., 2024). The ratio of negative cohesion to positive cohesion influenced community stability, with positive interactions potentially destabilizing it by positive feedback loops among taxonomic groups that support mutual adaptability (Bannon et al., 2022), while negative interactions enhanced stability by partitioning niches to enable the coexistence of multiple species (Wu et al., 2024). Peroxide treatments enhanced the stability and complexity of bacterial networks (Figures 3C, D), supporting the theory that more complex communities are more structurally stable (Cornell et al., 2023; Macarthur, 1955; Wang et al., 2023).

Fungal community displayed different patterns compared to bacteria. Fungal networks exhibited weakened negative interactions under calcium peroxide, indicating that fungal community tended to cooperate to acquire resources and adapt to environmental changes (Zhu et al., 2024). This finding was closely linked to the decrease in available nutrients and the increase in pH of soil microbial habitats caused by calcium peroxide. It is worth noting that the fungal stability declined under peroxide treatments (Figure 3G), verifying that the increasing positive interactions disrupted community stability (Bannon et al., 2022). Additionally, lower stability may indirectly demonstrate that peroxides could exert a disinfection and sterilization effect on certain fungi and reduce redundancy of fungal community.

Furthermore, the enhanced bacteria-fungi interactions under calcium peroxide suggest that these communities may respond more effectively to environmental changes, thereby maintaining soil ecological functions (Li et al., 2025). The reduced positive correlation ratio of interdomain interactions under peroxides reflected the improvement of connections between bacterial and fungal communities, while also highlighting the equal importance of cooperation and competition between interkingdom species (Deveau et al., 2018; Tao et al., 2025). Meanwhile, this finding accordingly indicated that long-term peanut cultivation may lead to an increase in positive connections, potentially disrupting the ecological balance of the microbial kingdoms (Li et al., 2022). Additionally, the interkingdom network exhibited various topological features, including higher average degree, increased connectance, and shorter average path length under calcium peroxide, supporting the hypothesis that calcium peroxide enhanced the interaction strength between bacteria and fungi, improved the resistance to external disturbance, and increased the efficiency of information and nutrient transmission.

### Association of microbial networks with soil properties and crop yield and the key environmental drivers

The significant increase in peanut productivity under calcium peroxide treatment, likely reflected its influence on soil environment and microbial community. Previous studies have shown that lime application can change soil pH and shape microbial communities, thereby effectively alleviating the soil obstacles and increasing crop yield (Bossolani et al., 2020; Lu et al., 2023; Yang et al., 2024). And our research found that calcium peroxide may exert a similar effect to lime. As anticipated, there was a strong correlation between peanut yield and microbial networks, with the stability, negative cohesion, and complexity of bacterial community playing a crucial role in regulating peanut yield (Figures 5C, D). Additionally, calcium peroxide altered the microbial community structure by increasing soil pH, given that pH was the most significant factor influencing the microbiome (Duan et al., 2025; Zhong et al., 2023). Peroxides increased soil pH and enhanced bacterial stability, which were beneficial for inhibiting pathogen invasion and promoting plant growth (Li X. G. et al., 2023). The pathogen disrupted the balance between reproductive and nutritional growth of peanut, causing nutrients to be preferentially allocated to the stems and leaves, which resulted in a decrease in pod yield (Luo et al., 2024; Zhou et al., 2023). In our study, the decrease in *Fusarium* and plant pathogen under peroxides indicated that the growth and reproduction of pathogen were partially inhibited, which contributed to peanut growth.

Bacterial community stability was observed to be the most important driver of the peanut yield in all treatments. The strengthened connections of microbial community, particularly the negative interactions, enhanced the complexity and stability of the community (Cui et al., 2025). Stable microbial community relies on more interactions to cope with pathogens and environmental stress (Huang et al., 2024; Li X. G. et al., 2023), and it tends to enhance soil multifunctionality (Long et al., 2025; Zhang et al., 2024). Consequently, the stability of bacterial community should be regarded as a key factor closely associated with crop productivity (Chen et al., 2024). In our study, calcium peroxide primarily modulated the microbial community structure by increasing soil pH. Furthermore, calcium peroxide and hydrogen peroxide could improve the soil environment by increasing soil oxygen availability and directly eliminating harmful organisms via oxygen free radicals, thereby stimulating microbial community and boosting microbial activity (Abd Elhady et al., 2021). This could explain why hydrogen peroxide treatment significantly enhanced the stability of bacterial communities and slightly increased peanut yield.

**Figure 6:**
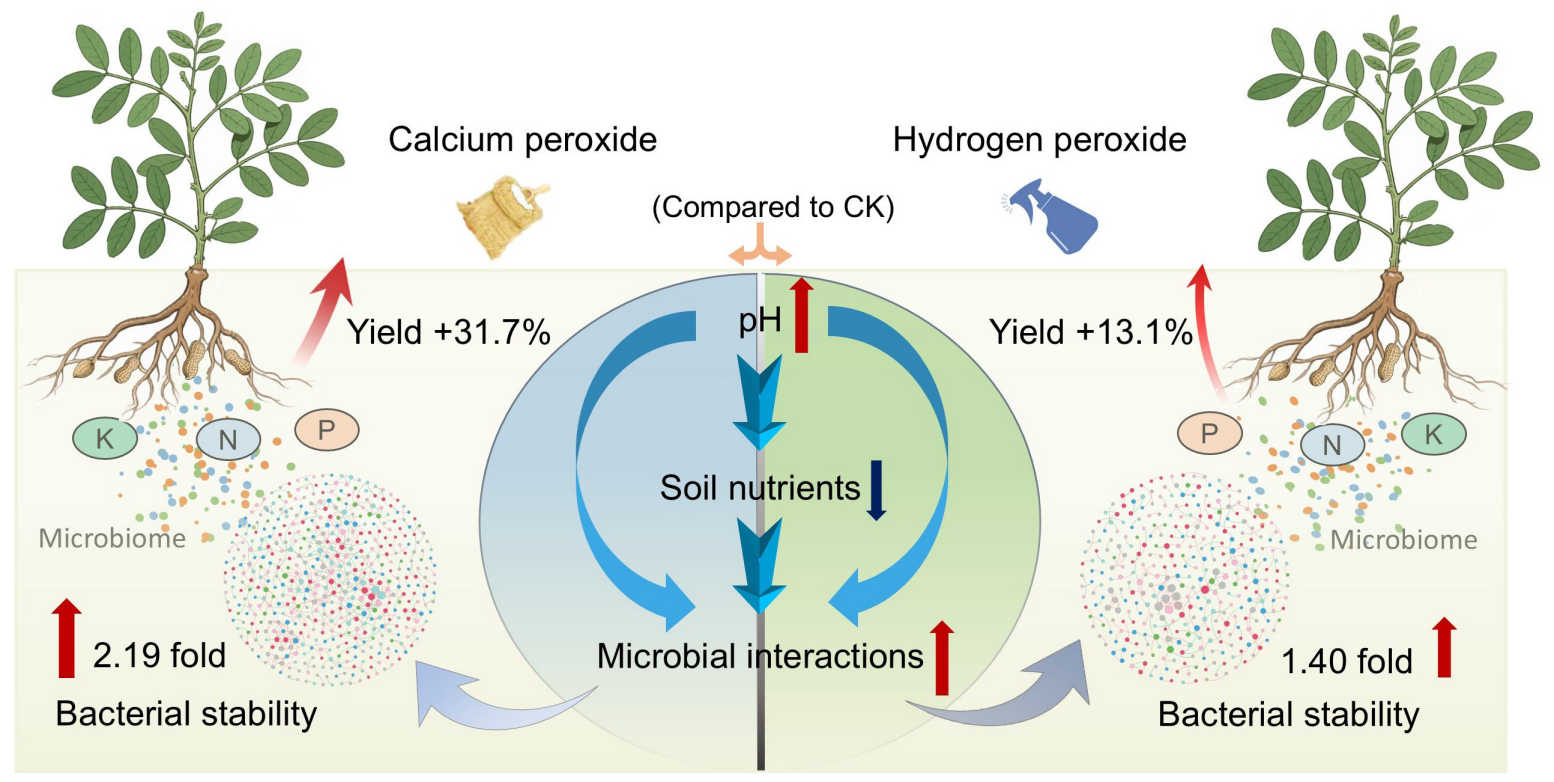
A conceptual model showing the potential microbial mechanisms underlying the short-term peroxide addition that enhances the interaction of soil microbial community, thereby promoting peanut growth.

This study indicated that peroxide application increased soil pH and enhanced microbial interactions, which were closely associated with the increase of peanut yield (Figure 6). In contrast, the short-term application effect of calcium peroxide is superior to that of hydrogen peroxide. In this light, our results have important implications for maintaining a healthy soil microbial community through the application of calcium peroxide, which may be necessary for improving crop productivity. However, it should be noted that our study only investigated the effects of peroxides on the overall interactions of microbial communities, which may underestimate the role of certain key microbial groups. Additionally, practical applications should warrant consideration of the long-term effects of peroxides, particularly calcium accumulation and the excessive consumption of organic matter by long-term oxidation. Further studies should elucidate the underlying microbial mechanisms and potential positive ecosystem functional effects of peroxide application, clarify the causal relationships among soil pH, microbial community structure, and peanut yield, and thereby provide the germplasm and theoretical basis for the bioremediation of peanut-planting soils.

## Conclusion

This study revealed that short-term peroxide application greatly affected soil physicochemical properties, microbial community, as well as peanut yield. Our results indicated that short-term calcium peroxide application significantly increased soil pH but reduced soil nutrient availability. Peroxides application significantly affected fungal community structure, while it rarely affected bacteria community. The relative abundance of *Fusarium* and plant pathogen decreased under peroxide treatments. Furthermore, peroxides application strengthened the network interactions of bacteria and enhanced the stability and complexity of bacterial community, which may improve resistance to pathogen. Calcium peroxide application significantly increased peanut yield, which was mainly attributed to the enhanced bacterial stability resulting from the increase in soil pH. Overall, our study provided a specific and feasible reference for relieving soil obstacles for long-term cultivation of peanut and supporting sustainable peanut production in acidic soils.

## Data Availability

The datasets presented in this study can be found in online repositories. The names of the repository/repositories and accession number(s) can be found below: https://www.ncbi.nlm.nih.gov/, accession numbers PRJNA1311041 and PRJNA1311036.
