# Supplementary material for: Short-term calcium peroxide application promotes soil microbial interactions to improve peanut yield in acidic soils
Source: Front Microbiol. 2026 Mar 30;17:1780622. doi: 10.3389/fmicb.2026.1780622 (PMC13075855; doi:10.3389/fmicb.2026.1780622)
Supplement: Supplementary file 1 [file Data_Sheet_1.docx]

**Supplementary material**

**Journal name:** Frontiers in Microbiology

**Title**: Short-term calcium peroxide application promotes soil microbial interactions to im·prove peanut yield in acidic soils

**Names of authors**: Yunfei Yu ^1,2^, Xiaoxue Li ^1,2^, Azhar Sohail Shahzad ^1,2^, Shunyao Zhuang ^1,2*^

**Complete postal address (es) or affiliations**:

^1^ State Key Laboratory of Soil and Sustainable Agriculture, Institute of Soil Science, Chinese Academy of Sciences, Nanjing 211135, P. R. China

^2^ University of the Chinese Academy of Sciences, Beijing 10049, P. R. China

**Full telephone, Fax number, E-mail, and present address of the corresponding author**:

^*^Corresponding author: Prof. Shunyao Zhuang

Tel.: +86-25-86881114

Fax: +86-25-86881000

E-mail: [syzhuang@issas.ac.cn](mailto:syzhuang@issas.ac.cn)

Present address: 298 Chuangyou Rd., Kirin St, Jiangning District, Nanjing 211135, China

**This file includes:** Figure S1 to S4**;** Table S1 to S4

**Figure captions**

**Figure S1** Temporal dynamics of bacterial and fungal α-diversity (Chao1 index) in different treatments (A, B). Different letters represent the significant difference at *p* < 0.05 by Duncan’s test. ns: not significant. Treatment: CK, control without peroxide application; CP, 0.2% calcium peroxide application; HP, 1% hydrogen peroxide application.

**Figure S2** PCoA of bacterial and fungal β-diversity based on Bray-Curtis distance (A, B). Treatment: CK, control without peroxide application; CP, 0.2% calcium peroxide application; HP, 1% hydrogen peroxide application.

**Figure S3** Relative abundance of bacterial (A) and fungal (B) genera in different treatments (n=3). Treatment: CK, control without peroxide application; CP, 0.2% calcium peroxide application; HP, 1% hydrogen peroxide application.

**Figure S4** Spearman correlation diagram presenting the relationships between soil physicochemical properties and microbial community composition of bacterial and fungal phyla (A, B).

**Figure S1**


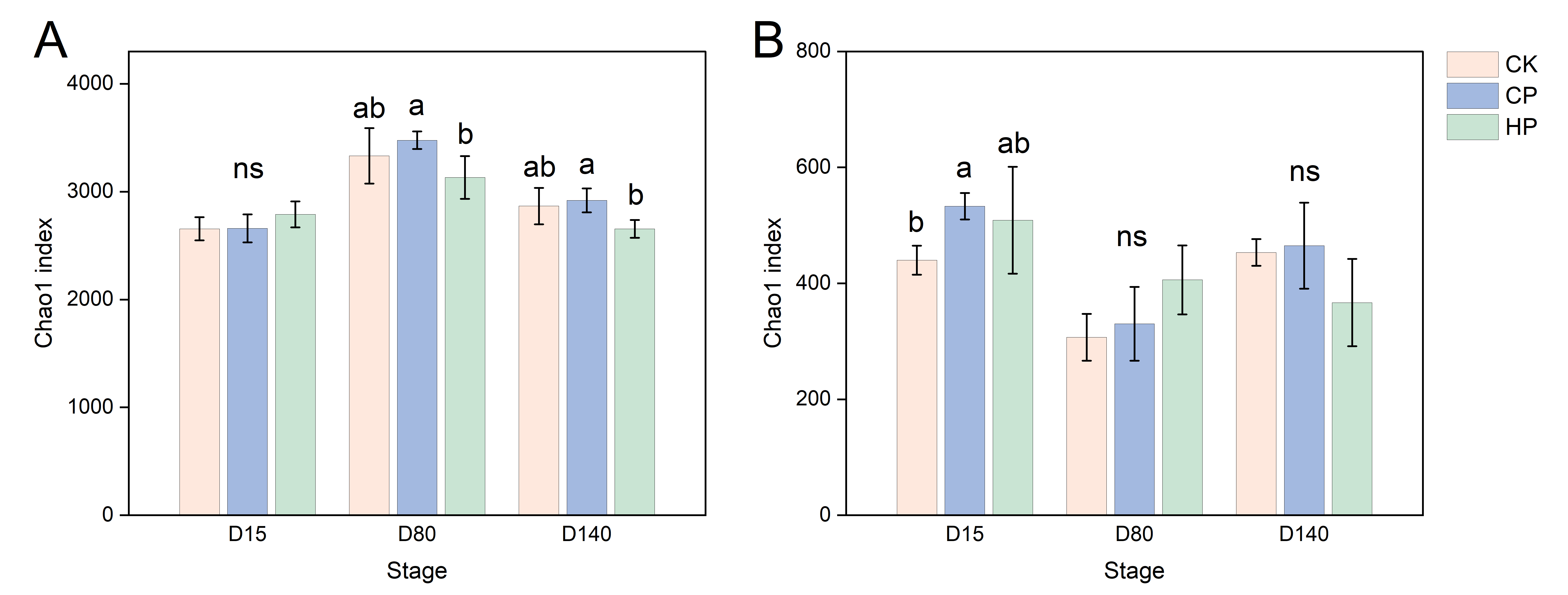


**Figure S2**


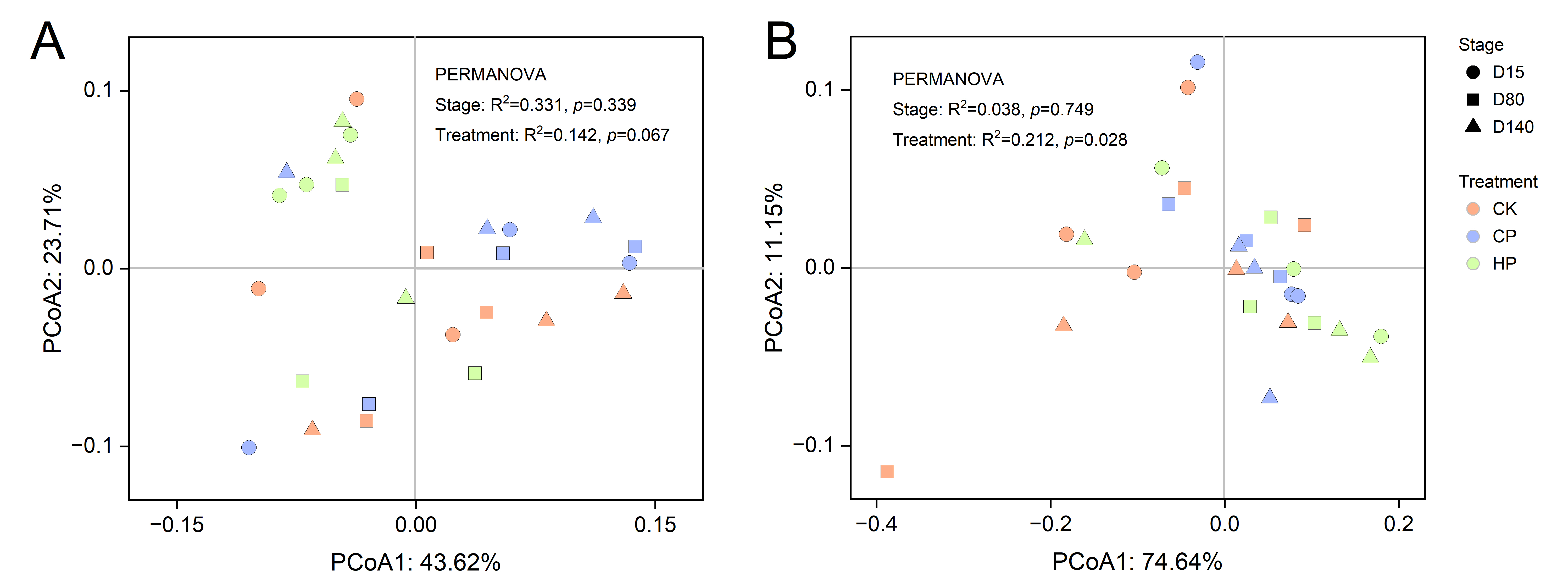


**Figure S3**


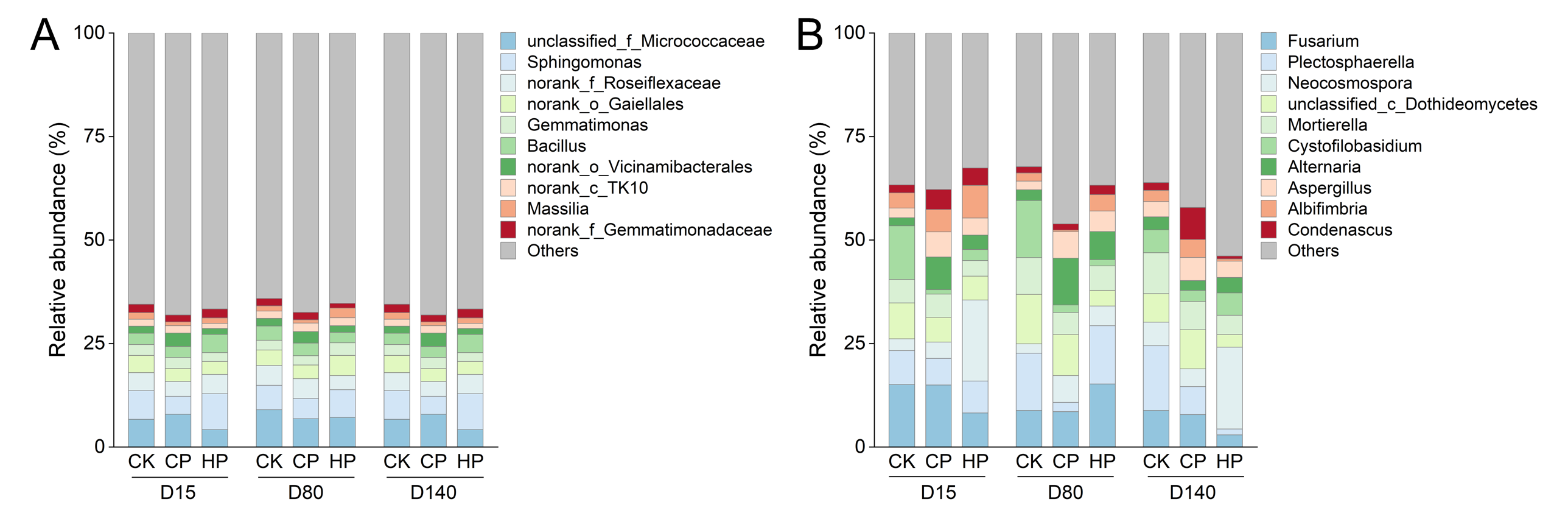


**Figure S4**


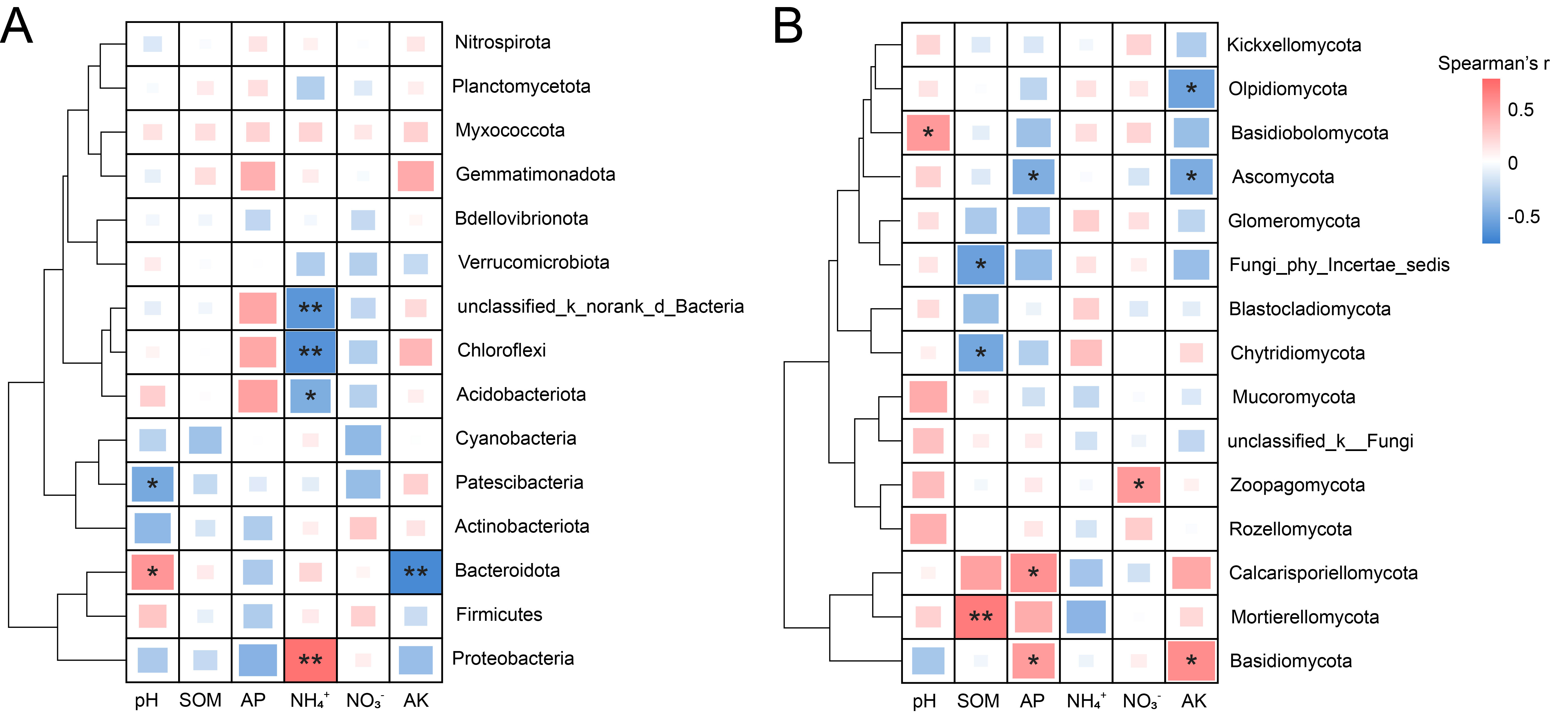


**Table captions**

**Table S1** Soil physicochemical properties under different treatments. Different letters indicate significant differences (*p* < 0.05) between treatments.

**Table S2** The topological properties of soil bacterial networks under different treatments.

**Table S3** The topological properties of soil fungal networks under different treatments.

**Table S4** The topological properties of soil bacteria-fungi networks under different treatments.

**Table S1**

| Treatment | CK | CP | HP |
| --- | --- | --- | --- |
|  | D15 | | |
| pH | 6.00±0.07b | 6.75±0.07a | 6.61±0.08a |
| SOM (g kg^-1^) | 8.96±1.58a | 10.34±2.31a | 8.69±2.04a |
| NH_4_^+^ (mg kg^-1^) | 3.29±0.72a | 2.01±0.64b | 2.30±0.24ab |
| NO_3_^-^ (mg kg^-1^) | 12.13±3.93a | 8.62±1.71a | 7.91±1.93a |
| Olsen-P (mg kg^-1^) | 56.74±5.68a | 43.64±13.3ab | 32.23±4.10b |
| Available K (mg kg^-1^) | 370.5±60.8a | 261.5±24.1b | 288.5±62.6ab |
|  | D80 | | |
| pH | 6.15±0.08b | 6.62±0.03a | 6.33±0.16ab |
| SOM (g kg^-1^) | 8.37±1.38a | 9.53±2.24a | 8.31±1.76a |
| NH_4_^+^ (mg kg^-1^) | 1.57±0.32ab | 1.00±0.16b | 1.75±0.39a |
| NO_3_^-^ (mg kg^-1^) | 5.57±0.98a | 3.46±1.02a | 4.83±1.15a |
| Olsen-P (mg kg^-1^) | 53.46±10.7a | 42.55±14.1a | 34.47±6.33a |
| Available K (mg kg^-1^) | 372.2±59.0a | 256.6±20.1b | 281.6±21.2b |
|  | D140 | | |
| pH | 5.99±0.30b | 6.53±0.04a | 6.31±0.15ab |
| SOM (g kg^-1^) | 9.96±1.63a | 9.55±1.87a | 8.71±1.64a |
| NH_4_^+^ (mg kg^-1^) | 1.22±0.13b | 1.75±0.55ab | 2.38±0.63a |
| NO_3_^-^ (mg kg^-1^) | 8.80±0.89a | 4.90±0.32b | 5.74±1.01b |
| Olsen-P (mg kg^-1^) | 59.81±6.49a | 43.18±15.4a | 41.45±8.02a |
| Available K (mg kg^-1^) | 396.2±24.0a | 256.6±47.1b | 269.8±24.2b |

**Table S2**

| Network indexes | CK | CP | HP |
| --- | --- | --- | --- |
| Total nodes | 115 | 477 | 333 |
| Total links | 70 | 543 | 241 |
| Positive correlation ratio | 74.3% | 67.6% | 69.3% |
| Negative correlation ratio | 25.7% | 32.4% | 30.7% |
| Average degree | 1.21 | 2.28 | 1.45 |
| Connectance | 0.011 | 0.005 | 0.004 |
| Network diameter | 2 | 28 | 6 |
| Average path length | 1.07 | 8.65 | 1.78 |
| Modularity | 0.97 | 0.92 | 0.98 |
| Heterogeneity | 0.40 | 0.75 | 0.57 |

**Table S3**

| Network indexes | CK | CP | HP |
| --- | --- | --- | --- |
| Total nodes | 102 | 107 | 101 |
| Total links | 140 | 164 | 141 |
| Positive correlation ratio | 95.7% | 96.3% | 99.3% |
| Negative correlation ratio | 4.3% | 3.7% | 0.7% |
| Average degree | 2.75 | 3.07 | 2.79 |
| Connectance | 0.027 | 0.029 | 0.028 |
| Network diameter | 3 | 4 | 3 |
| Average path length | 1.24 | 1.33 | 1.20 |
| Modularity | 0.81 | 0.85 | 0.82 |
| Heterogeneity | 0.94 | 0.77 | 0.97 |

**Table S4**

| Network indexes | CK | CP | HP |
| --- | --- | --- | --- |
| Total nodes | 255 | 255 | 255 |
| Total links | 1096 | 1717 | 1053 |
| Positive correlation ratio | 60.1% | 50.7% | 51.8% |
| Negative correlation ratio | 39.9% | 49.3% | 48.2% |
| Network diameter | 7 | 7 | 9 |
| Modularity | 0.46 | 0.38 | 0.48 |
